# Detailed functional analysis of two clinical glucose-6-phosphate dehydrogenase (G6PD) variants, G6PD_Viangchan_ and G6PD_Viangchan_ _+_ _Mahidol_: Decreased stability and catalytic efficiency contribute to the clinical phenotype

**DOI:** 10.1016/j.ymgme.2016.03.008

**Published:** 2016-06

**Authors:** Usa Boonyuen, Kamonwan Chamchoy, Thitiluck Swangsri, Naowarat Saralamba, Nicholas P.J. Day, Mallika Imwong

**Affiliations:** a Department of Molecular Tropical Medicine and Genetics, Faculty of Tropical Medicine, Mahidol University, Bangkok 10400, Thailand; b Mahidol-Oxford Tropical Medicine Research Unit, Faculty of Tropical Medicine, Mahidol University, Bangkok 10400, Thailand; c Centre for Tropical Medicine, Nuffield Department of Medicine, University of Oxford, Oxford, United Kingdom

**Keywords:** Glucose-6-phosphate dehydrogenase, G6PD deficiency, Variants, Steady state kinetics, Thermostability

## Abstract

Deficiency of glucose-6-phosphate dehydrogenase (G6PD) is an X-linked hereditary genetic defect that is the most common polymorphism and enzymopathy in humans. To investigate functional properties of two clinical variants, G6PD_Viangchan_ and G6PD_Viangchan_ _+_ _Mahidol_, these two mutants were created by overlap-extension PCR, expressed in *Escherichia coli* and purified to homogeneity. We describe an overexpression and purification method to obtain substantial amounts of functionally active protein. The *K*_M_ for G6P of the two variants was comparable to the *K*_M_ of the native enzyme, whereas the *K*_M_ for NADP^+^ was increased 5-fold for G6PD_Viangchan_ and 8-fold for G6PD_Viangchan_ _+_ _Mahidol_ when compared with the native enzyme. Additionally, *k*_cat_ of the mutant enzymes was markedly reduced, resulting in a 10- and 18-fold reduction in catalytic efficiency for NADP^+^ catalysis for G6PD_Viangchan_ and G6PD_Viangchan_ _+_ _Mahidol_, respectively. Furthermore, the two variants demonstrated significant reduction in thermostability, but similar susceptibility to trypsin digestion, when compared with the wild-type enzyme. The presence of NADP^+^ is shown to improve the stability of G6PD enzymes. This is the first report indicating that protein instability and reduced catalytic efficiency are responsible for the reduced catalytic activity of G6PD_Viangchan_ and G6PD_Viangchan_ _+_ _Mahidol_ and, as a consequence, contribute to the clinical phenotypes of these two clinical variants.

## Highlights

• Recombinant human G6PD enzymes were expressed in *E. coli* and substantial amount of enzymes were obtained.
• Detailed biochemical characterization of G6PD enzymes was performed.
• Purified G6PD variants showed a significant decrease in catalytic efficiency and protein stability.
• Data obtained provide further knowledge in phenotype-genotype relationship of G6PD deficiency.

## 1 Introduction

Glucose-6-phosphate dehydrogenase (G6PD, E.C. 1.1.1.49) is a metabolic enzyme that catalyzes the oxidation of glucose-6-phosphate (G6P) to 6-phosphogluconolactone, a key step in the pentose phosphate pathway, and produces the reduced form of nicotinamide adenine dinucleotide phosphate (NADPH). NADPH is necessary for protection of the cells against oxidative stress [1], [2], [3]. It is particularly important in red blood cells where G6PD activity is the only source of NADPH and where lacking a nucleus no new G6PD can be synthesized. G6PD deficiency is a hereditary genetic defect, which is the most prevalent polymorphism and enzymopathy in humans [4]. The gene encoding G6PD is located at the q28 locus on the X-chromosome. Therefore, clinical features are more prominent in the male population. While homozygous females are relatively rare, heterozygous females exhibit a range of enzyme activities due to partial inactivation of one X-chromosome early in embryogenesis (lyonization). G6PD deficiency is common and responsible for a variety of clinical conditions, affecting around 400 million people worldwide [5]. Clinical manifestations of G6PD deficiency include favism, hemolytic anemia, chronic non-spherocytic hemolytic anemia (CNSHA), spontaneous abortions and neonatal hyperbilirubinemia resulting in neonatal kernicterus that can lead to death [6], [7], [8]. WHO working groups have classified G6PD variants based on enzyme activity into five classes with thresholds of 10–60% as a defined level of G6PD activity [4]. More than 180 G6PD deficiencies have been identified at the DNA level and over 400 variants described based on biochemical properties [9], [10], [11]. Variation in the enzyme activity of G6PD deficiencies has an impact on malaria treatment, because antimalarials such as primaquine, sulfanilamide and sulfadoxine have been observed to cause hemolysis in G6PD deficient individuals [12]. This hemolytic toxicity has raised significant concern because of the widespread prevalence of G6PD deficiencies in malaria endemic regions [13], [14], [15]. Importantly, use of antimalarials is problematic because the hemolytic anemia observed in individuals with G6PD deficiencies is variable. The clinical hemolytic episode can range from mild and self-limiting to severe and even fatal, depending on exposure and the G6PD genotype. As a consequence, information regarding the relationship between G6PD genotypes and G6PD activity is crucial.

Biochemical characterizations, enzyme kinetics and structural studies have been extensively used to investigate the mechanisms underlying human G6PD deficiencies. Expression, purification and characterization of several single mutation G6PD variants have been described [16], [17], [18], [19]. It was observed that mutations located in close proximity to and important for binding with substrates (G6P and NADP^+^) and subunit contact sites have a direct effect on enzyme activity [20], [21], [22], [23]. On the other hand, some mutations affect protein folding and reduce protein stability [18], [24], [25]. The only double mutant that has been thoroughly characterized was G6PD A^−^ (Val68Met + Asn126Asp). This variant influences protein folding, whereas the catalytic efficiency is not affected [24], [26]. Additionally, G6PD A^−^ showed poor refolding activity, indicating that protein folding is impaired.

G6PD_Viangchan_, which has an amino acid alteration at residue 291 from valine to methionine, was first discovered in a Laotian immigrant in 1988 [27] and is also prevalent in Thailand and Cambodia [14], [28]. It has been classified as a Class II deficiency based on biochemical characterization of a partially purified enzyme. The partially purified enzyme from red blood cells displays altered enzymatic activities, affecting binding affinity for substrates and an inhibitor. However, no further investigations detailing the impact of the mutation have been carried out for G6PD_Viangchan_. G6PD variants identified are mostly single mutations; however, G6PD multiple mutations were also found with lower frequency when compared to a single mutation. In the latest update of the G6PD database, 12 double, 1 triple and 1 quadruple mutants were reported [11]. Recently, another two double mutants in the Thai population were described, which are G6PD_Viangchan_ _+_ _Mahidol_ and G6PD_Viangchan_ _+_ _Union_ [29]. Following partial purification from red blood cells these double mutations exhibited severe enzyme deficiency with a remaining activity of < 10%. The combined G6PD_Viangchan_ _+_ _Mahidol_ mutant showed an approximately 10-fold reduction in enzyme activity when compared with the single mutation variants. Full characterization of these natural variants is, therefore, required to fully understand the molecular mechanisms underlying the observed clinical manifestations. In addition, information obtained will provide insight into the relationship between genotype and phenotypic characteristics of G6PD variants.

In this study, we report methods for overexpression and purification of G6PD proteins. Two natural mutants, G6PD_Viangchan_ and G6PD_Viangchan_ _+_ _Mahidol_, were expressed, and steady state kinetics, thermostability and trypsin digestion were carried out to biochemically characterize these two mutants.

## 2 Materials and methods

### 2.1 Construction of recombinant G6PD and site-directed mutagenesis

The G6PD gene was amplified from human cDNA using Phusion® High Fidelity DNA polymerase (Thermo Scientific) with G6PD forward and reverse primers (Table 1). Two restriction enzyme sites were introduced, *Eco*RI and *Xho*I, in the forward and reverse primers, respectively (underlined). PCR products were gel purified and digested with *Eco*RI and *Xho*I (New England Biolabs). The digested amplicon was ligated into the pET28a expression vector with an N-terminal His-tag and transformed into the expression host BL21 (DE3). Three natural variants, G6PD_Mahidol_ (Gly163Ser), G6PD_Viangchan_ (Val291Met) and G6PD_Viangchan_ _+_ _Mahidol_ (Val291Met + Gly163Ser), were generated by an overlap-extension PCR method as described previously by Ho et al. using primers listed in Table 1 [30]. All constructs were verified by bidirectional DNA sequencing and restriction digestion to confirm that the desired recombinant plasmids were obtained.

### 2.2 Expression and purification of G6PD enzymes

A single colony of BL21 (DE3) harboring the desired plasmid was inoculated in 5 ml of LB medium containing 50 μg/ml kanamycin and incubated at 37 °C with 250 rpm shaking overnight. The overnight cultures were inoculated into fresh 1 l of LB medium in the presence of 50 μg/ml kanamycin at a dilution of 1:100 and grown at 37 °C with 250 rpm shaking until the absorbance at 600 nm (OD_600_) reached 0.6–0.8. The cultures were then induced with isopropyl β-d-thiogalactoside (IPTG, Merck) at a final concentration of 1 mM and were further incubated at 20 °C with 180 rpm shaking for 20 h before being harvested by centrifugation at 1000 ×* g* for 15 min.

Cell pellets were resuspended in lysis buffer (20 mM sodium phosphate, pH 7.4, 300 mM NaCl, 10 mM imidazole) and disrupted by sonication. The cell lysate was centrifuged at 20,000 ×* g* for 45 min at 4 °C, and the supernatant was collected. Subsequently, the supernatant was incubated with pre-equilibrated TALON Metal Affinity Resin (BD Biosciences) in lysis buffer at 4 °C for at least 1 h. The unbound proteins were removed with wash buffer (20 mM sodium phosphate, pH 7.4, 300 mM NaCl, 20 mM imidazole). The elution of G6PD protein was accomplished with the elute buffer (20 mM sodium phosphate, pH 7.4, 300 mM NaCl, and 40–400 mM imidazole). Imidazole was removed by buffer exchange with 20 mM Tris-HCl, pH 7.5 containing 10% glycerol using Amicon Ultra centrifugal filter devices (Millipore). Next, the protein was loaded onto an anion exchange column (HiTrap™ QXL, GE Healthcare). The unbound proteins were washed with 20 mM Tris-HCl pH 7.5 and the G6PD protein was eluted with a gradient concentration of 0.1–1.0 M NaCl in 20 mM Tris-HCl pH 7.5. Finally, NaCl was removed by buffer exchange with 20 mM Tris-HCl pH 7.5 containing 10% glycerol to stabilize the enzyme. Proteins from each purification step were analyzed by 12% SDS-PAGE stained with Coomassie blue (Sigma-Aldrich). The protein concentration was determined by the Bradford assay [31]. The purified enzyme was stored in the presence of 10 μM NADP^+^ at − 20 °C.

### 2.3 Western blot analysis

Western blot analysis was performed to confirm the expression of recombinant G6PD. Purified proteins were loaded onto a 12% SDS polyacrylamide gel, separated by electrophoresis and transferred to a polyvinylidene difluoride (PVDF) membrane. The membrane was blocked with phosphate-buffered saline (PBS) containing 5% skimmed milk at room temperature for 1 h and then incubated at 4 °C overnight with an anti-human G6PD antibody (Pierce), at a dilution of 1:3000 in the same solution. After washing 4 times with PBS containing 0.05% Tween 20 (PBST) for 15 min each, the membrane was incubated with a horseradish peroxidase (HRP)-conjugated anti-mouse secondary antibody (Pierce) diluted 1:2500 in PBST. Prior to development, the membrane was washed 4 times with PBST for 15 min each. Finally, the proteins were detected using the ECL system (Merck Millipore) and the membrane was visualized using an ImageQuant LAS4000 mini system (GE Healthcare Life Sciences).

### 2.4 Measurement of steady-state kinetic parameters

G6PD activity was measured spectrophotometrically by monitoring the reduction of NADP^+^ at 340 nm using a UV-2700 UV-VIS spectrophotometer (Shimadzu). The standard activity assay was performed in a cuvette with a final volume of 1 ml. The reaction mixture contained 50 mM Tris-HCl, pH 8.0, 0.01 M MgCl_2_, 200 μM NADP^+^ and 500 μM G6P. The reaction was initiated with the addition of the enzyme. Determination of kinetic constants was performed by varying the concentration of one substrate (2.5–500 μM for NADP^+^ and 5–500 μM for G6P) and fixing the concentration of the other substrate at saturating concentration (200 μM for NADP^+^ and 500 μM for G6P). Each reaction was conducted four times and the initial linear measurement was used for determining the slope of the initial velocity. Data from the spectrophotometer were exported to Excel to calculate the rate of product formation and were expressed as micromole of NADPH produced per minute per milligram protein (μmol/min/mg) as calculated using the extinction coefficient for NADPH at 340 nm (6220 M^− 1^ cm^− 1^). Steady-state kinetic parameters, *K*_M_, *k*_cat_ and *V*_max_, were obtained by fitting the collected data to the Michaelis-Menten equation using GraphPad Prism software (GraphPad Software).

### 2.5 Circular dichroism (CD) analysis

Far UV-CD spectra of the G6PD enzymes (0.35 mg/ml) were recorded using a Jasco spectrometer, model J-815, with a 1 mm path-length. The measurements were carried out at 25 °C. The spectra were collected over a wavelength range of 190–260 nm at a scan rate of 50 nm/min. Three scans were averaged for each protein sample and the buffer was subtracted.

The thermostability of G6PD enzymes was also assessed by CD analysis. Bound NADP^+^ was removed from purified enzyme by buffer exchanged with 20 mM Tris-HCl pH 7.5 and adjusted a concentration of G6PD protein to 0.2 mg/ml. The stability of WT and each variant was monitored by following the change in the CD signal at 222 nm while varying the temperature from 20 to 80 °C at a rate of 3 °C/min.

### 2.6 Thermostability tests

For the thermostability test, bound NADP^+^ was removed from the purified enzyme by buffer exchanged with 20 mM Tris-HCl pH 7.5 and the enzyme concentration was adjusted to 0.2 mg/ml. The enzyme was incubated in the presence of different concentrations of NADP^+^ (0, 1, 10, 100 and 1,000 μM) for 20 min at temperatures ranging from 25 to 65 °C and then cooled down to 4 °C in a Thermocycler (Eppendorf). Residual activity of the enzyme was determined and expressed as a percentage of the activity for the same enzyme incubated at 25 °C.

### 2.7 Trypsin digestion

Bound NADP^+^ was removed from the purified enzyme as aforementioned and the enzyme concentration was adjusted to a concentration of 0.2 mg/ml. Susceptibility of G6PD to trypsin was investigated in the presence of different concentrations of NADP^+^ (1, 10 and 100 μM). Trypsin (0.5 mg/ml) was added to enzyme samples and incubated at 25 °C. The residual enzyme activity was examined at time intervals (5–120 min) and expressed as a percentage of the activity for the same enzyme without incubation.

## 3 Results

### 3.1 Construction, expression and purification of recombinant human G6PD enzymes

**Fig. 1:**
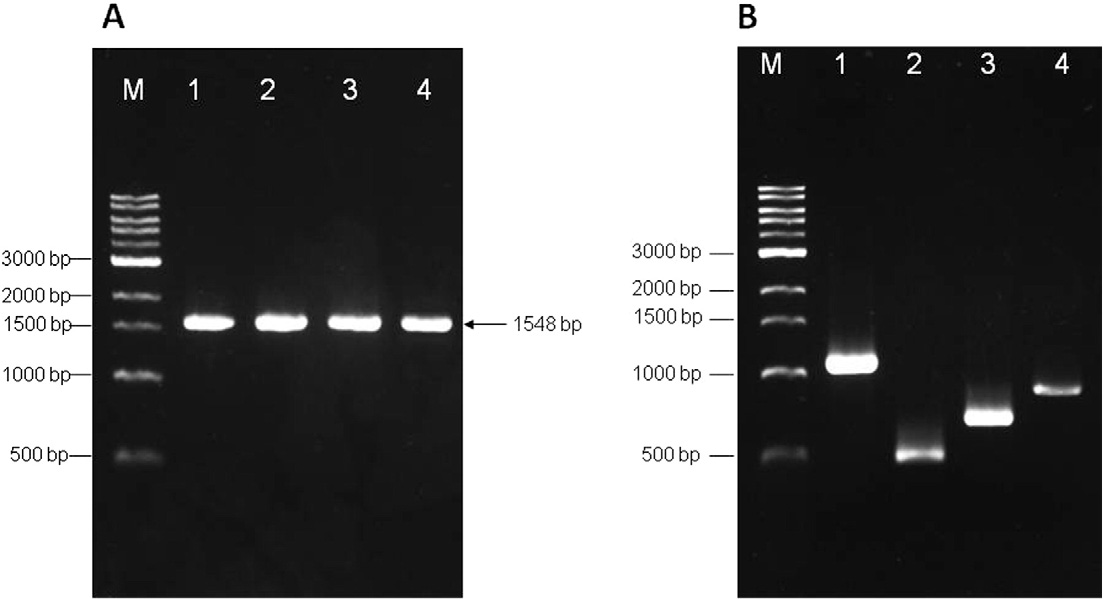
Agarose gel electrophoresis. A) Full-length PCR products. Lane M, DNA ladder; lane 1, G6PD WT; lane 2, G6PD_Mahidol_; lane 3, G6PD_Viangchan_; and lane 4, G6PD_Viangchan_ _+_ _Mahidol_. B) Products of the first round of overlap-extension PCR. Lane M, DNA ladder; lane 1, G6PD_Mahidol_ fragment 1; lane 2, G6PD_Mahidol_ fragment 2; lane 3, G6PD_Viangchan_ fragment 1; and lane 4, G6PD_Viangchan_ fragment 2. Fragments 1 and 2 were used as templates for the amplification of the full-length product of G6PD variants.

**Fig. 2:**
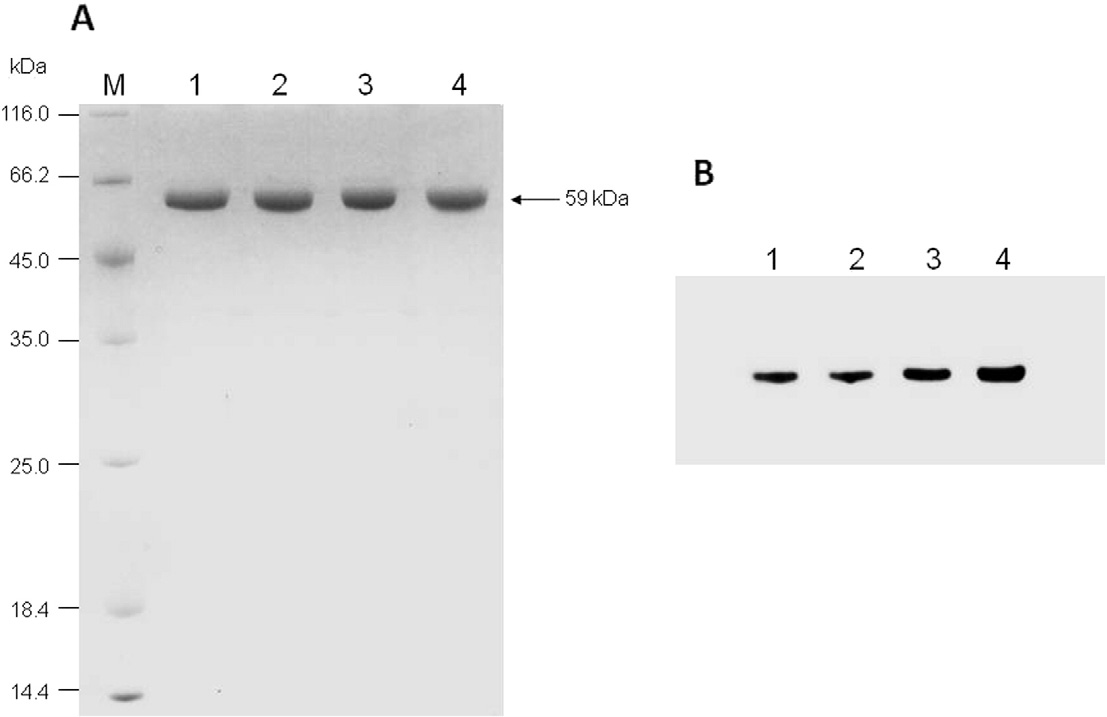
SDS-PAGE and western blot analysis of recombinant human G6PD proteins. A) SDS-PAGE of purified enzymes: lane M, molecular mass marker proteins; lane 1, G6PD WT; lane 2, G6PD_Mahidol_; lane 3, G6PD_Viangchan_; and lane 4, G6PD_Viangchan_ _+_ _Mahidol_. The arrow indicates the expected size of recombinant human G6PD. B) Western blot analysis of purified recombinant human G6PD proteins: lane 1, G6PD WT; lane 2, G6PD_Mahidol_; lane 3, G6PD_Viangchan_; and lane 4, G6PD_Viangchan_ _+_ _Mahidol_. Proteins were immunodetected using an anti-human G6PD antibody.

The human G6PD gene was amplified by PCR from human cDNA with the expected size of 1548 bp (Fig. 1A), and cloned into the pET28a expression vector at the *Eco*RI and *Xho*I sites to yield pET28a-G6PD WT. pET28a-G6PD WT was used as a template for overlap-extension PCR to create two single mutants, G6PD_Viangchan_ and G6PD_Mahidol_ (Fig. 1B). Fragments obtained from overlap extension PCR were used to amplify the full-length genes that were subsequently cloned into the pET28a expression vector at the same cloning sites. Thereafter, pET28a-G6PD_Viangchan_ was used as a template to create the double mutant G6PD_Viangchan_ _+_ _Mahidol_. Recombinant G6PD was expressed in *Escherichia coli* BL21 (DE3) and purified using cobalt-affinity and anion exchange columns. SDS-PAGE analysis of WT and the three mutants is shown in Fig. 2A. To confirm the expression of human G6PD, western blot analysis was performed using an anti-human G6PD antibody (Fig. 2B). The details of each purification step are shown in Table 2.

### 3.2 Steady state kinetic parameters

**Fig. 3:**
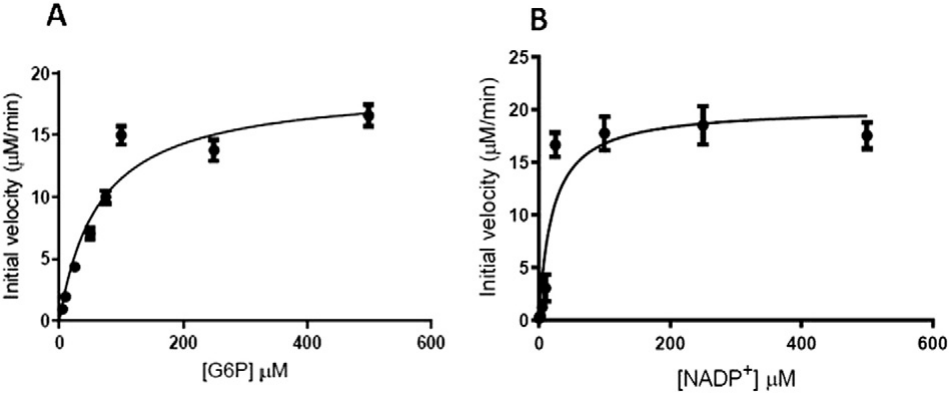
Representative kinetic plots of G6PD_Mahidol_ for A) G6P and B) NADP^+^.

Steady state kinetic parameters were determined for three clinical variants and G6PD WT, where G6PD WT and G6PD_Mahidol_ were included in the study as controls (Table 3). Representative kinetic plots showing the Michaelis-Menten constants of G6PD_Mahidol_ for G6P and NADP^+^ were shown in Fig. 3. The catalytic activities (*k*_cat_) of WT and G6PD_Mahidol_ are comparable to previous reports [18]. The two clinical mutants G6PD_Viangchan_ and G6PD_Viangchan_ _+_ _Mahidol_ showed ~ 2-fold decrease in catalytic activity when compared with the native enzyme. Although the affinity for G6P was only slightly affected by these two mutations, the *K*_M_ for NADP^+^ was significantly disrupted for the G6PD_Viangchan_ and G6PD_Viangchan_ _+_ _Mahidol_ mutants with *K*_MNADP^+^_ values of 34.1 ± 2.9 and 55.9 ± 8.9 μM, respectively, which is approximately 5- and 8-fold greater than the WT enzyme. As a consequence, the catalytic efficiency for these two clinical variants is decreased by 10- and 18-fold for G6PD_Viangchan_ and G6PD_Viangchan_ _+_ _Mahidol_, respectively, when compared with the value of the WT enzyme.

### 3.3 CD analysis

**Fig. 4:**
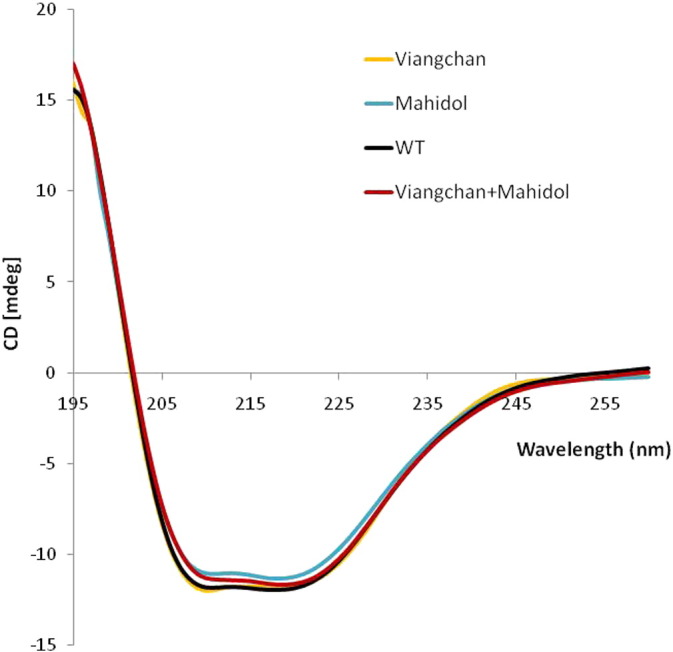
Circular dichroism (CD) spectra of recombinant human G6PD enzymes: WT, G6PD_Mahidol_, G6PD_Viangchan_ and G6PD_Viangchan_ _+_ _Mahidol_. The protein concentration was 0.35 mg/ml in 20 mM Tris-HCl pH 7.5.

**Fig. 5:**
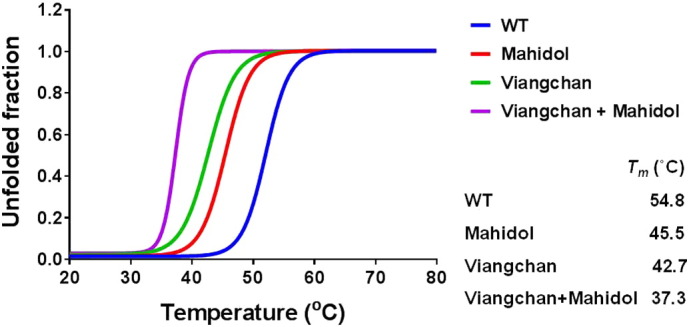
Changes in the CD signal at 222 nm for G6PD WT and the three natural variants as the temperature is increased.

Since the catalytic efficiency of the two natural variants, G6PD_Viangchan_ and G6PD_Viangchan_ _+_ _Mahidol_, was markedly reduced, the effect of a conformational change caused by the mutations was investigated. The secondary structure of native and the three G6PD mutants was examined by CD analysis in the far UV region (190–260 nm) (Fig. 4). CD spectra of the three natural variants shared a similar pattern to that of the native enzyme, indicating that the mutations did not cause an alteration to the secondary structure of the enzyme. However, the thermal denaturation of each variant was different, as assessed by measuring the secondary structure at 222 nm. The melting temperature (temperature at which half of secondary structure is unfolded) was 54.8, 45.5, 42.7, 37.3 °C for G6PD WT, G6PD_Mahidol_, G6PD_Viangchan_ and G6PD_Viangchan_ _+_ _Mahidol_, respectively (Fig. 5).

### 3.4 Thermostability

**Fig. 6:**
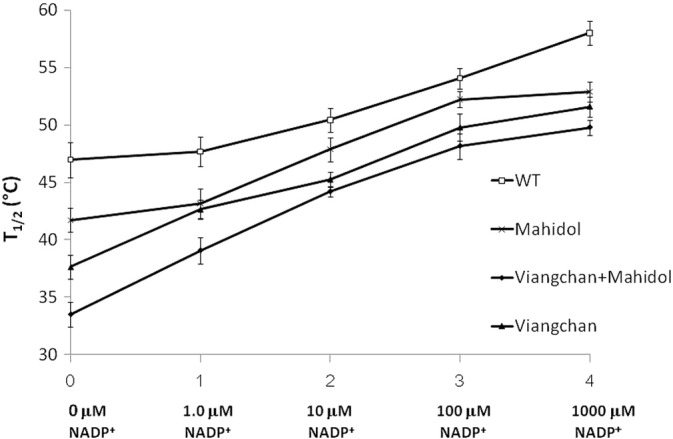
Thermostability analyses of recombinant G6PD enzymes in the absence and presence of different concentrations of NADP^+^. *T*_1/2_ (temperature at which the enzyme loses 50% of its activity) is plotted against the NADP^+^ concentration.

It is well documented that the presence of a low concentration of NADP^+^ is required for long-term stability of the enzyme [10], [23], [32]. Therefore, thermostability of WT and the three G6PD variants was further elucidated in the absence and presence of different concentrations of NADP^+^ (0, 1, 10, 100 and 1,000 μM) (Fig. 6). The WT enzyme exhibited greater stability than the G6PD variants in the absence or presence of NADP^+^, and the stability of all enzyme variants increased with increasing concentration of NADP^+^. *T*_1/2_ (temperature at which the enzyme loses 50% of its activity) of all enzymes was increased 11–16 °C in the presence of 1,000 μM NADP^+^. G6PD_Viangchan_ _+_ _Mahidol_ was the least stable enzyme with a *T*_1/2_ of 33.5 °C, which is 13.5 °C lower than the WT enzyme (*T*_1/2_ = 47 °C) in the absence of NADP^+^, indicating that this variant is structurally unstable.

### 3.5 Trypsin digestion

**Fig. 7:**
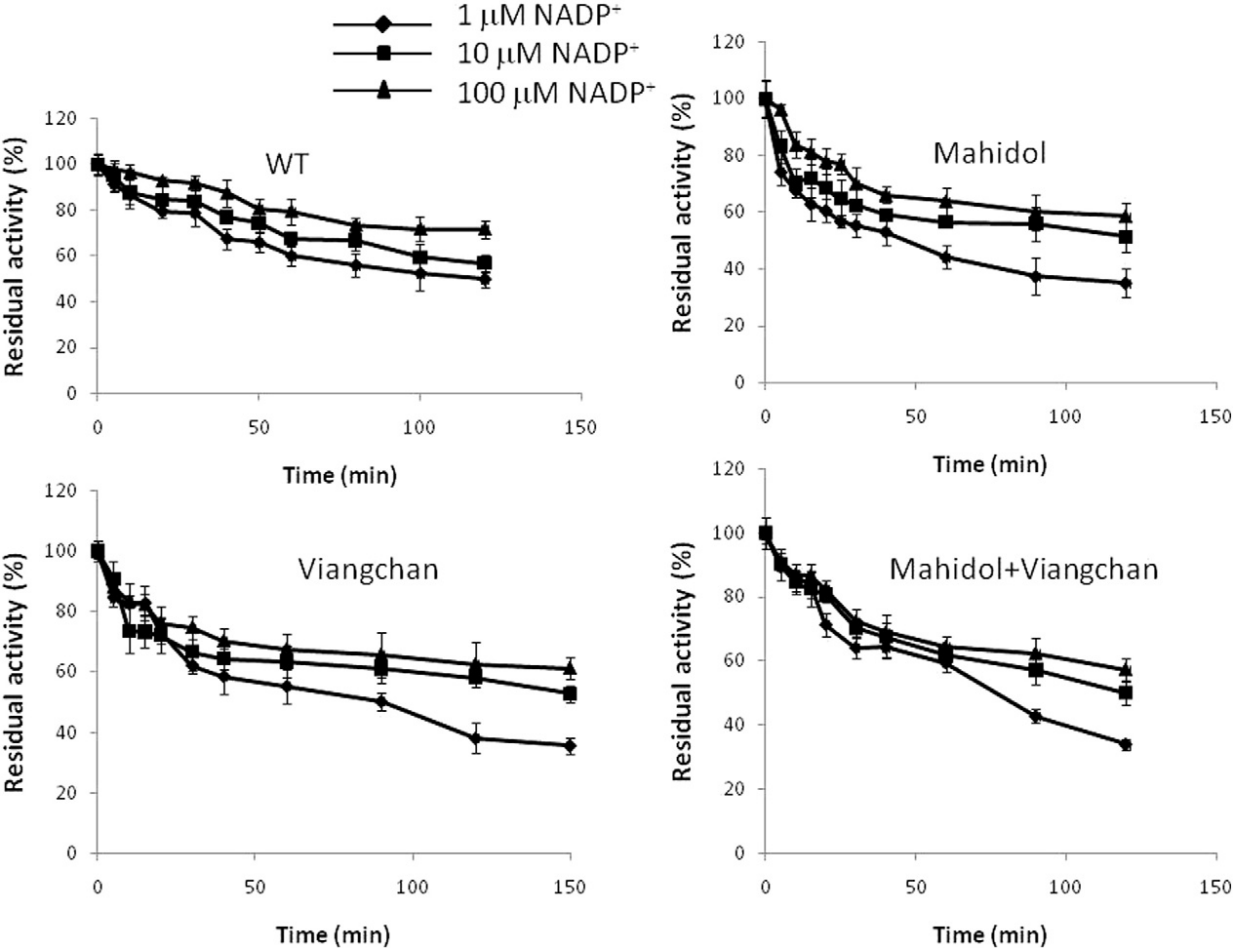
Susceptibility of recombinant G6PD enzymes to trypsin digestion in the presence of different concentrations of NADP^+^.

The susceptibility of G6PD WT and the three variants to trypsin digestion was assessed at 25 °C in the presence of different concentrations of NADP^+^ (1, 10 and 100 μM) (Fig. 7). All three variants displayed comparable susceptibility to trypsin digestion in comparison with the native enzyme. After 2 h of digestion in the presence of 10 μM NADP^+^, which is the physiological concentration, G6PD WT retained ~ 57% of its original activity, whereas all three natural mutants retained ~ 50% of their activity. The addition of NADP^+^ showed a protective effect against trypsin digestion, especially for the G6PD variants where the presence of NADP^+^ has a slightly more prominent impact.

## 4 Discussion

The two main causes of reduced G6PD activity in humans are either a decrease in catalytic activity or a reduction in the number of active G6PD molecules. The decrease in catalytic efficiency is dependent on the position of the mutation. Reduction in the number of active enzyme molecules can be caused by protein instability, inability to form stable dimers, impairment of protein folding and problems with mRNA splicing [18]. As a consequence, many studies have focused on the protein level to examine the molecular mechanisms regarding the reduced G6PD activity observed in individuals [16], [17], [18], [19], [24], [25], [33], [34].

In the present study, we thoroughly investigated the functional properties of two natural G6PD variants, G6PD_Viangchan_ and G6PD_Viangchan_ _+_ _Mahidol_. G6PD WT and G6PD_Mahidol_ were included in this study as controls. The bacterial expression system has been widely used to produce recombinant proteins from various organisms because bacteria are easy to handle and time- and cost-efficient. Previously, expression of human G6PD was performed in various organisms, e.g., G6PD WT in *E. coli*, G6PD_Plymouth_ and G6PD_Mahidol_ in *E. coli* and G6PD_Suwalki_ in yeast [17], [18], [35]. The recombinant G6PD purified from a bacterial expression system exhibited indistinguishable characteristics to the G6PD purified from human red blood cells. Furthermore, the recombinant G6PD made from bacteria also displayed a longer shelf-life and greater stability, retaining 100% activity when stored at 4 °C for over a year [35]. Therefore, in this study, we chose bacterial expression to produce G6PD proteins. G6PD variants were cloned into the pET28a expression vector and expressed in bacteria BL21 (DE3). The amount of G6PD proteins obtained was substantial and adequate for biochemical characterization; however, the G6PD_Viangchan_ and G6PD_Viangchan_ _+_ _Mahidol_ yields were relatively low when compared with the yield obtained for the WT protein. Low protein yields for these two variants could be due to their low stability, as it was observed during purification that G6PD_Viangchan_ and G6PD_Viangchan_ _+_ _Mahidol_ aggregated easily even in the presence of 10% glycerol and NADP^+^. In this study, all G6PD proteins purified were His-tagged and they were characterized without removal of the His-tagged because it was shown earlier that additional His-tagged did not affect the catalytic activity and stability of the G6PD enzymes [36].

**Fig. 8:**
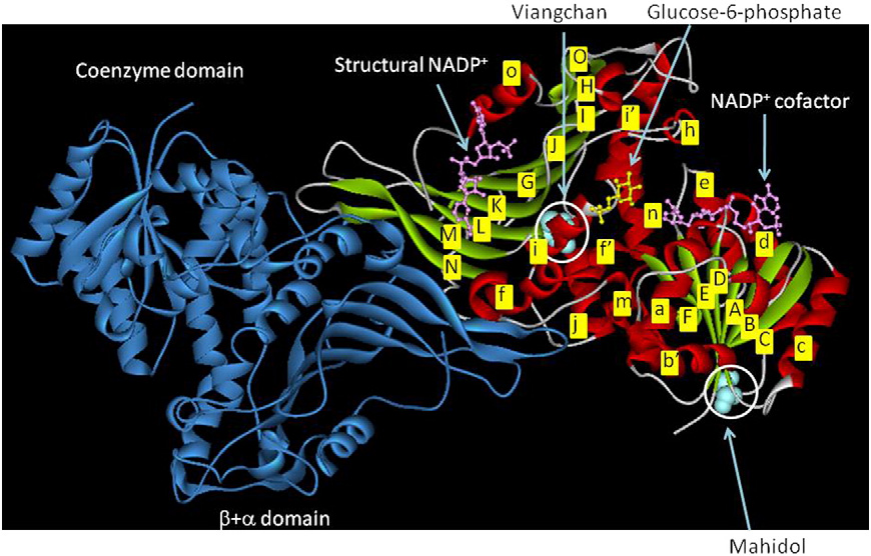
Three-dimensional structure of G6PD_Mahidol_ _+_ _Viangchan_ dimer. Helices and sheet strands of A subunit are shown in red and green, respectively. B subunit is shown in blue. Each of the secondary structure elements is labeled. The location of each mutation is highlighted by the white circle. The figure was created with Discovery Studio Visualizer – Accelrys.

Detailed biochemical characterization was performed for four G6PD enzymes. Steady-state kinetic parameters of G6PD WT and G6PD_Mahidol_ are in good agreement with previous reports [18], [25], [33]. As shown in Fig. 8, G6PD_Mahidol_ has an amino acid mutation at residue 163 from glycine to serine, sitting at the front of the strand βE, on the surface of the enzyme, and in close proximity to the base of the coenzyme binding domain. Mutation at this residue causes steric hindrance, thereby affecting the conformation of the protein. G6PD_Mahidol_ displayed only a small decrease in catalytic activity when compared with the WT enzyme ([18] and this study). However, this variant showed a reduction in thermostability in the absence or presence of NADP^+^, which could explain the reduced enzyme activity observed in red blood cells of G6PD deficient individuals. On the other hand, the Val291Met mutation of G6PD_Viangchan_ is on the large β + α domain, αj, of the protein structure. Although this mutation is not close to the structural NADP^+^ binding site nor substrate binding site, a partially purified enzyme from erythrocytes showed decreased enzyme activity and alteration in affinity towards substrates [27]. Similar findings were observed for G6PD_Union_ and G6PD_Andalus_, where small structural changes lead to a significant reduction in the catalytic efficiency [33]. Therefore, it is of interest to extensively investigate the effects of the Val291Met mutation on kinetic properties. In this study, purified recombinant G6PD_Viangchan_ showed a 2-fold reduction in *k*_cat_ when compared with the native enzyme. The *K*_M_ for G6P of this mutation was comparable to G6PD WT whereas the *K*_M_ for NADP^+^ was 5-fold higher. The purified recombinant G6PD_Viangchan_ in our study exhibited a small difference in binding affinity for G6P from that reported for a partially purified enzyme from red blood cells. The discrepancies in *K*_M_ for G6P could arise from different assay systems and interference present in a partially purified enzyme. The catalytic efficiency of G6PD_Viangchan_ was about 10-fold lower than the native enzyme. Novel double mutant G6PD variants, G6PD_Viangchan_ _+_ _Mahidol_ and G6PD_Viangchan_ _+_ _Union,_ were reported from Thailand in 2013 [29]. Partially purified enzymes of these double mutants showed a significant decrease in enzyme activity, motivating our efforts to characterize the biochemical effects of such double mutations. The double mutant G6PD_Viangchan_ _+_ _Mahidol_ exhibited a > 2-fold decrease in *k*_cat_ when compared with that of G6PD WT. Although the *K*_M_ for G6P of this mutant was similar to the *K*_M_ of G6PD WT, for NADP^+^ the value was raised by 8-fold causing a significant reduction in the catalytic efficiency by ~ 20-fold with respect to the native enzyme. The remarkable reduction in enzyme catalytic efficiency observed in this study for G6PD_Viangchan_ and G6PD_Viangchan_ _+_ _Mahidol_ could provide an explanation for the reduced enzyme activity observed in erythrocytes.

In addition to reduced catalytic efficiency, protein instability can also explain the reduced enzyme activity in G6PD deficiency individuals. Structural and biochemical studies of G6PD enzymes have indicated that structural NADP^+^ is essential for the stability and maintenance of enzyme activity [10], [23], [32]. The presence of structural NADP^+^ was shown to increase protein stability for many G6PD enzyme variants, e.g., G6PD_Union_, G6PD_Andalus_, G6PD_Plymouth_ and G6PD_Nashville_ [18], [25], [33]. Although the location of the mutation for both G6PD_Mahidol_ and G6PD_Viangchan_ is not in close proximity to the dimer interface and binding sites of structural and coenzyme NADP^+^ and the G6P substrate, the effects of these mutations on protein stability were elucidated, since it has been shown that G6PD_Mahidol_ causes a local conformational change and affects backbone folding, resulting in protein instability [18]. The instability of G6PD_Mahidol_, G6PD_Viangchan_ and G6PD_Viangchan_ _+_ _Mahidol_ variants is unlikely to cause a disruption to the secondary structure as they have similar CD spectra to the CD spectrum of the WT enzyme. Therefore, the thermal denaturation of WT and the three G6PD mutants was assessed by CD analysis. The melting temperature (*T*_m_), the temperature at which half of the secondary structure is unfolded, was 54.8 °C for WT, 45.5 °C for G6PD_Mahidol_, 42.7 °C for G6PD_Viangchan_ and 37.3 °C for G6PD_Viangchan_ _+_ _Mahidol_. The melting temperature of G6PD WT from this study is in good agreement with the value of 55 °C reported previously [37]. G6PD_Mahidol_, G6PD_Viangchan_ and G6PD_Viangchan_ _+_ _Mahidol_ are highly unstable as they lost 50% of their secondary structure below 50 °C. In particular, for G6PD_Viangchan_ _+_ _Mahidol_ the melting temperature is 15 °C lower than the *T*_m_ of the native enzyme. The difference in melting temperature between native and variant enzymes was further confirmed by carrying out a thermostability test of the enzymes in the absence and presence of different concentrations of NADP^+^. The presence of NADP^+^ was shown to increase thermostability for all G6PD proteins, in agreement with previous reports for G6PD WT and G6PD_Mahidol_ [18]. G6PD_Viangchan_ _+_ _Mahidol_ was the least stable G6PD variant studied here with a *T*_1/2_ of 33.5 °C in the absence of NADP^+^ and 49.8 °C in the presence of 10 μM NADP^+^. These *T*_1/2_ values are much lower than the native enzyme under the same conditions with values of 47 °C and 58 °C in the absence and presence of 10 μM NADP^+^, respectively. G6PD_Viangchan_ also exhibited significantly reduced thermostability when compared with G6PD WT, but the effect is most pronounced for G6PD_Viangchan_ _+_ _Mahidol_.

Defective protein folding can also reduce protein stability and usually leads to accelerated degradation. Several G6PD mutations have been shown to be more susceptible to protease degradation [25], [37]. Susceptibility to trypsin digestion was investigated for G6PD enzymes. No significant increase in susceptibility to trypsin digestion was observed for the three natural G6PD variants studied here. The presence of NADP^+^ has been shown to counteract the susceptibility to proteolysis in a concentration-dependent manner. The results obtained here were in agreement with a previous report for G6PD_Mahidol_ where no significant differences in susceptibility to trypsin digestion were observed when compared with the G6PD WT [18]. However, it remains inconclusive because all G6PD enzymes were in the fully folded native form and the experiments were carried out *in vitro*, which are different from physiological conditions where protein degradation occurs in a proteasome. In addition, proteases present in human cells may have different protein quality controls from the trypsin used.

## 5 Conclusions

In summary, the reduced activity of G6PD variants observed in red blood cells may originate from several causes. Reduced catalytic efficiency and protein stability are clearly the major causes of clinical enzyme deficiency observed in individuals carrying G6PD_Viangchan_ and G6PD_Viangchan_ _+_ _Mahidol_. From detailed biochemical characterization, we report here, for the first time, that G6PD_Viangchan_ exhibited a 10-fold decrease in catalytic efficiency and this variant also showed lower thermostability when compared with the native enzyme. This result further supports the classification of this variant as a Class II G6PD deficiency. Moreover, the double mutant enzyme, G6PD_Viangchan_ _+_ _Mahidol,_ displayed an even greater loss in catalytic efficiency (18-fold) with a significant reduction in thermostability when compared with G6PD WT. This double mutant, therefore, should be classified as a severe G6PD deficiency. However, clinical data is also required to precisely allocate it to a Class I or Class II G6PD deficiency. Thus, functional characterization of both G6PD variants has provided valuable information describing the molecular mechanisms underlying the severity of clinical manifestations.

## Conflict of interest statement

The authors state that there is no conflict of interest.

## Author contributions

Conceived and designed the experiments: UB and MI. Performed the experiments: UB, KC, TS and NS. Contributed reagents/materials/analysis tools: UB, ND and MI. Wrote the paper: UB, ND and MI.

## Figures and Tables

**Table 1:** List of primers used in DNA cloning and site-directed mutagenesis.

Primers	Sequence
G6PD_F	5′ GGATCCGAATTCATGGCAGAGCAGG 3′
G6PD_R	5′ GGTGCTCGAGTCAGAGCTTGTGGGG 3′
Mahidol_F	5′ CGAGTCCTGCATGAGCCAGATAAGCTGGAA 3′
Mahidol_R	5′ TTCCAGCTTATCTGGCTCATGCAGGACTCG 3′
Viangchan_F	5′ GATGAGAAGGTCAAGATGTTGAAATGCATC 3′
Viangchan_R	5′ GATGCATTTCAACATCTTGACCTTCTCATC 3′

**Table 2:** Purification of recombinant human G6PD enzymes.

Construct	Purification step	Total protein (mg)	Total activity (IU)	Specific activity (IU/mg)	Yield (%)	Purity (%)
WT	Crude extract	49.7	2200	44.3	100	19.4%
Affinity column	20.1	1965	97.8	89.3	42.9%
Anion exchange column	7.8	1780	228.2	80.1	100%
G6PD_Mahidol_	Crude extract	43.6	1775	40.7	100	28.8%
Affinity column	19.2	1438	74.9	81.0	53.1%
Anion exchange column	8.9	1256	141.1	70.8	100%
G6PD_Viangchan_	Crude extract	39.3	1532	39.0	100	36.3%
Affinity column	17.8	1108	62.2	72.3	57.9%
Anion exchange column	7.4	795	107.4	51.9	100%
G6PD_Viangchan_ _+_ _Mahidol_	Crude extract	32.7	1226	37.5	100	47.5%
Affinity column	16.7	894	53.5	72.9	67.7%
Anion exchange column	6.9	545	79.0	44.4	100%

**Table 3:** Steady-state kinetic parameters of recombinant human G6PD enzymes.

Construct	*k*_cat_ (s^− 1^)	*K*_MG6P_ (μM)	*K*_MNADP^+^_ (μM)	*k*_cat_/*K*_MG6P_ (μM^− 1^ s^− 1^)	*k*_cat_/*K*_MNADP^+^_ (μM^− 1^ s^− 1^)
WT	247 ± 9	47.8 ± 4.2	7.2 ± 1.8	5.2 ± 0.7	34.3 ± 2.9
G6PD_Mahidol_	224 ± 8	46.9 ± 5.4	5.9 ± 0.8	4.8 ± 0.5	38.1 ± 2.6
G6PD_Viangchan_	116 ± 4	56.3 ± 4.8	34.1 ± 2.9	2.0 ± 0.1	3.4 ± 0.2
G6PD_Viangchan_ _+_ _Mahidol_	104 ± 4	54.3 ± 5.1	55.9 ± 8.9	1.9 ± 0.1	1.9 ± 0.1
